# 1-[4-(Difluoro­meth­oxy)phen­yl]-*N*-(3,4-dimeth­oxy­phen­yl)-1*H*-1,2,4-triazole-3-carboxamide

**DOI:** 10.1107/S1600536810029661

**Published:** 2010-08-11

**Authors:** Yu-Guang Wang, Guo-Bo Huang, Bing-Chun Zhu

**Affiliations:** a College of Biological and Environmental Engineering, Zhejiang University of Technology, Hangzhou 310014, People’s Republic of China; b School of Pharmaceutical and Chemical Engineering, Taizhou University, Taizhou 317000, People’s Republic of China; c Zhejiang University of Technology, Hangzhou 310014, People’s Republic of China, Zhejiang Research Institute of Chemical Industry, Hangzhou 310023, People’s Republic of China

## Abstract

Two crystallographically independent mol­ecules, *A* and *B*, with similar conformations are present in the asymmetric unit of the title compound, C_18_H_16_F_2_N_4_O_4_. In mol­ecule *A*, the plane of the 1,2,4-triazole ring is tilted relative of the 4-difluoro­meth­oxy-substituted and the 3,4-dimeth­oxy-substituted benzene rings by 6.5 (2) and 16.4 (1)°, respectively. The –CHF_2_ group is twisted away from the plane of the benzene ring, with a dihedral angle between the O—C bond of the OCHF_2_ group and the plane of the adjacent phenyl ring of 38.6 (3)°. The corresponding parameters for mol­ecule *B* are  7.7 (1), 9.5 (2) and 25.2 (2)°. In both mol­ecules, the conformations are stabilized by intra­molecular N—H⋯N and C—H⋯O hydrogen bonds. There are also C—H⋯π contacts between the methyl groups and the benzene rings, and π–π stacking inter­actions between the benzene rings of adjacent parallel *A* mol­ecules [centroid–centroid distance = 3.8942 (17) Å]. π–π inter­actions are also observed between the triazole ring and one of the benzene rings of parallel *B* mol­ecules [centroid–centroid distance = 3.7055 (16) Å].

## Related literature

For the biological and pharmacological activity of 1,2,4-triazoles, see: Almasirad *et al.* (2004 ▶); Amir & Shikha (2004 ▶); Ibrahim (2009 ▶); Kalluraya *et al.* (1996 ▶); Kondo *et al.* (1992 ▶); Kanazawa *et al.* (1988 ▶); Labanauskas *et al.* (2004 ▶); Tozkoparan *et al.* (2007 ▶); Vlasova *et al.* (1971 ▶); Wahbi *et al.* (1995 ▶). For details of the synthesis, see: Drutkowski *et al.* (2002 ▶).
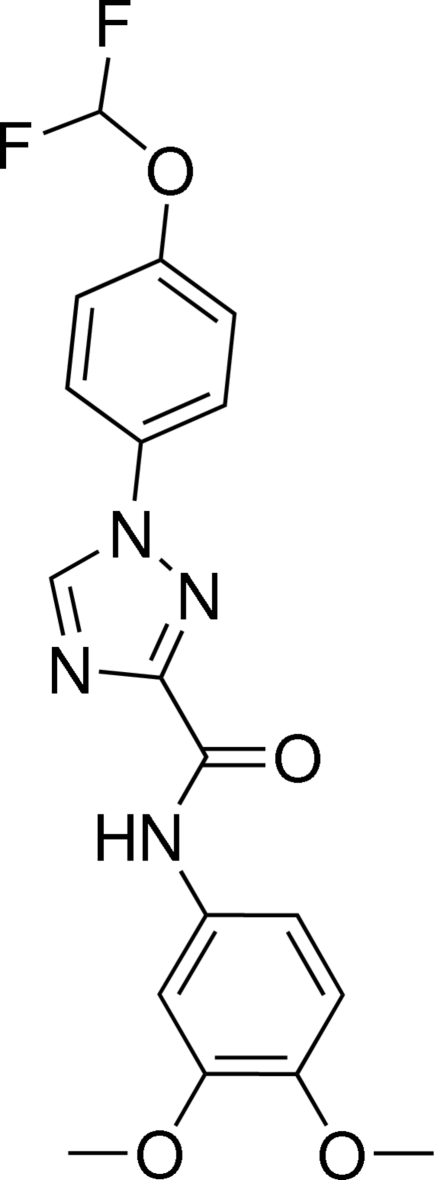


## Experimental

### 

#### Crystal data

C_18_H_16_F_2_N_4_O_4_
*M* *_r_* = 390.35
Triclinic, 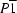

*a* = 9.4015 (19) Å
*b* = 12.138 (2) Å
*c* = 16.270 (3) Å
α = 77.345 (2)°
β = 88.040 (2)°
γ = 87.376 (2)°
*V* = 1809.1 (6) Å^3^
*Z* = 4
Mo *K*α radiation
μ = 0.12 mm^−1^
*T* = 294 K
0.45 × 0.39 × 0.31 mm

#### Data collection

Bruker APEXII CCD area-detector diffractometer
Absorption correction: multi-scan (*SADABS*; Sheldrick, 1996 ▶) *T*_min_ = 0.949, *T*_max_ = 0.965
13864 measured reflections
6692 independent reflections
4477 reflections with *I* > 2σ(*I*)
*R*_int_ = 0.020

#### Refinement

*R*[*F* ^2^ > 2σ(*F* ^2^)] = 0.050
*wR*(*F* ^2^) = 0.147
*S* = 1.02
6692 reflections
509 parameters
12 restraints
H-atom parameters constrained
Δρ_max_ = 0.48 e Å^−3^
Δρ_min_ = −0.34 e Å^−3^

### 

Data collection: *APEX2* (Bruker, 2004 ▶); cell refinement: *SAINT* (Bruker, 2004 ▶); data reduction: *SAINT*; program(s) used to solve structure: *SHELXS97* (Sheldrick, 2008 ▶); program(s) used to refine structure: *SHELXL97* (Sheldrick, 2008 ▶); molecular graphics: *SHELXTL* (Sheldrick, 2008 ▶); software used to prepare material for publication: *SHELXL97*.

## Supplementary Material

Crystal structure: contains datablocks global, I. DOI: 10.1107/S1600536810029661/zl2279sup1.cif

Structure factors: contains datablocks I. DOI: 10.1107/S1600536810029661/zl2279Isup2.hkl

Additional supplementary materials:  crystallographic information; 3D view; checkCIF report

## Figures and Tables

**Table 1:** Hydrogen-bond geometry (Å, °) *Cg*2 and *Cg*5 are the centroids of the C1–C6 and C19–C24 rings, respectively.

*D*—H⋯*A*	*D*—H	H⋯*A*	*D*⋯*A*	*D*—H⋯*A*
C20—H20*A*⋯O7	0.93	2.29	2.891 (3)	122
C6—H6*A*⋯O3	0.93	2.32	2.895 (3)	120
N1—H1*A*⋯N2	0.86	2.33	2.767 (3)	112
N5—H5*A*⋯N7	0.86	2.33	2.776 (3)	112
C11—H11*A*⋯O7^i^	0.93	2.51	3.406 (3)	162
C17—H17*A*⋯O7^i^	0.93	2.26	3.190 (3)	174
C8—H8*A*⋯O1^ii^	0.96	2.59	3.533 (3)	167
C31—H31*A*⋯O3^iii^	0.93	2.27	3.169 (3)	163
C8—H8*C*⋯*Cg*5	0.96	2.74	3.615 (3)	152
C25—H25*B*⋯*Cg*2^iv^	0.96	2.68	3.578 (3)	157

Symmetry codes: (i) 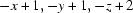; (ii) 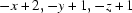; (iii) 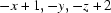; (iv) 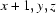.

**Fig. 1.**
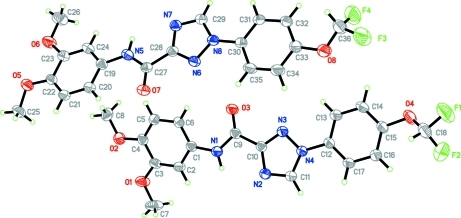
View of the title compound showing the atom numbering scheme and the ellipsoids at the 50% probability level.

**Fig. 2.**
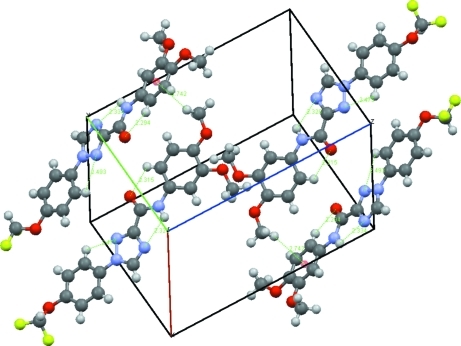
CH-π interactions and π-π stacking interactions.

## supplementary crystallographic information

### Comment

1,2,4-Triazoles and their derivatives have attracted our attention because of their diverse biological and pharmacological activities, such as anticonvulsant (Almasirad *et al.* 2004; Kanazawa *et al.* 1988), anticancer (Ibrahim 2009), antitubercular (Kalluraya *et al.* 1996; Vlasova *et al.* 1971), anti-inflammatory (Labanauskas *et al.*, 2004), and their herbicidal (Kondo *et al.* 1992) and analgesic properties (Amir & Shikha 2004; Tozkoparan *et al.* 2007). Also, antifungal activity of aromatic ethers possessing a 1*H*-1,2,4-triazole ring has been reported (Wahbi *et al.* 1995). We report here the crystal structure of one such 1,2,4-triazole (Fig. 1).

The asymmetric unit of the title compound contains two crystallographically independent molecules A and B, made up of C1 to C18 and C19 to C36, respectively. In both molecules, bond lengths and angles are within normal ranges. In the triazole ring of molecule A, the bond lengths of N3—C10 (1.312 (3) Å) and N2—C11 (1.316 (3) Å) indicate double bond character, while the distance of N2—C10 (1.357 (3) Å) is in the range for N—C single bonds; in the same way, the bond lengths of 1.314 (3) and 1.315 (3) Å point towards double bond character for N6—C28 and N7—C29, the bond length of N7—C28 (1.361 (3) Å) is indicative for an N—C single bond in molecule B. The O7 and O3 atoms are located in both molecules on the same side of the molecule (that of N6 and N3, respectively). In molecule A, the plane of the 1,2,4-triazole ring is tilted against that of the 4-difluoromethoxy-substituted (C12–C17) and the 3,4-dimethoxy-substituted (C1–C6) benzene rings by 6.5 (2)° and 16.4 (1)°, respectively. The CHF_2_ group is twisted away from the plane of the benzene ring, with a dihedral angle between the O—C bond of the OCHF_2_ group and the plane of the adjacent phenyl ring of 38.7 (3)°. In molecule B, the plane of the 1,2,4-triazole ring is tilted against that of the the 4-difluoromethoxy-substituted (C30–C35) and 3,4-dimethoxy-substituted (C19–C24) benzene rings by 7.7 (2)° and 9.5 (2)°, respectively. Its CHF_2_ group is also twisted away from the plane of the benzene ring with a dihedral angle between the O—C bond of the OCHF_2_ group and the plane of the adjacent phenyl ring of 25.2 (2)°. The two molecules are not coplanar with each other and exhibit torsion angles of 44.79 (8)° (between the C1–C6 benzene rings and C19–C24 benzene rings) and 11.6 (1)° (between the C12–C17 benzene rings and C30–C35 benzene rings), respectively.

The conformations of the two molecules are stabilized by intramolecular N—H···N hydrogen bonds and intra and intermolecular C—H···O hydrogen bonds (Table 1). There are also C—H···π contacts between the methyl groups and the benzene rings. The closest distance between the benzene rings of adjacent parallel A molecules is 3.33 Å indicating π–π stacking. Between parallel B molecules π–π interactions are observed between the triazole ring and one of the benzene rings (the closest distance is 3.32 Å). These weak interactions lead to the formation of a three-dimensional network as shown in Fig. 2.

### Experimental

The general procedure for the synthesis of the title compound is based on a literature procedure (Drutkowski *et al.*, 2002). 2-amine-*N*-(3,4-dimethoxy-phenyl)-2-[(4-difluoromethoxy- phenyl)hydrazono]acetamide (10 mmol), 1.5 ml of a 37%-solution of formaldehyde (20 mmol) and 0.1 g *p*-toluene sulfonic acid were heated to reflux in approximately 50 ml ethanol. The reaction was complete after 10 h (the reaction progress was controlled by TLC). The mixture was cooled to room temperature and the solvent was evaporated. The solid product was collected and recrystallized from 2-propanol. The yield of the reaction was 38%. Mp: 466-468K. MS (EI, 70 eV, Agilent 5975 inert mass selective detector): m/z 390 (M^+^, 100). IR (Bruker Vector 22 spectrometer): ν_max_ (cm^-1^) 3388, 3118, 1691, 1552, 1523, 1471, 1416,1222, 1124, 1031, 985, 841, 782, 639. ^1^H NMR (CDCl_3_, 400MHz, Bruker Avance spectrometer): δ 9.22(1H, s), 8.74 (1H, s), 7.85-7.42 (3H, m), 7.28-6.84 (4H, m), 6.75-6.39 (1H, t), 4.02 (3H, s), 3.94 (3H, s).

### Refinement

H atoms were placed in calculated positions with C—H = 0.93–0.98 Å, N—H = 0.86 Å, and refined in riding mode with *U*_iso_(H) = 1.5*U*_eq_(C) for methyl C, and  *U*_iso_(H) = 1.2*U*_eq_(C, N) for all other H atoms.

### Crystal data

**Table:**

C_18_H_16_F_2_N_4_O_4_	*Z* = 4
*M**_r_* = 390.35	*F*(000) = 808
Triclinic, *P*1	*D*_x_ = 1.433 Mg m^−^^3^
*a* = 9.4015 (19) Å	Mo *K*α radiation, λ = 0.71073 Å
*b* = 12.138 (2) Å	Cell parameters from 3656 reflections
*c* = 16.270 (3) Å	θ = 2.4–22.6°
α = 77.345 (2)°	µ = 0.12 mm^−^^1^
β = 88.040 (2)°	*T* = 294 K
γ = 87.376 (2)°	Block, colorless
*V* = 1809.1 (6) Å^3^	0.45 × 0.39 × 0.31 mm

### Data collection

**Table:**

Bruker APEXII CCD area-detector diffractometer	6692 independent reflections
Radiation source: fine-focus sealed tube	4477 reflections with *I* > 2σ(*I*)
graphite	*R*_int_ = 0.020
phi and ω scan	θ_max_ = 25.5°, θ_min_ = 2.4°
Absorption correction: multi-scan (*SADABS*; Sheldrick, 1996)	*h* = −11→11
*T*_min_ = 0.949, *T*_max_ = 0.965	*k* = −14→14
13864 measured reflections	*l* = −19→19

### Refinement

**Table:**

Refinement on *F*^2^	Primary atom site location: structure-invariant direct methods
Least-squares matrix: full	Secondary atom site location: difference Fourier map
*R*[*F*^2^ > 2σ(*F*^2^)] = 0.050	Hydrogen site location: inferred from neighbouring sites
*wR*(*F*^2^) = 0.147	H-atom parameters constrained
*S* = 1.02	*w* = 1/[σ^2^(*F*_o_^2^) + (0.0679*P*)^2^ + 0.5044*P*] where *P* = (*F*_o_^2^ + 2*F*_c_^2^)/3
6692 reflections	(Δ/σ)_max_ = 0.001
509 parameters	Δρ_max_ = 0.48 e Å^−^^3^
12 restraints	Δρ_min_ = −0.34 e Å^−^^3^

### Special details

**Table:**

Geometry. All esds (except the esd in the dihedral angle between two l.s. planes) are estimated using the full covariance matrix. The cell esds are taken into account individually in the estimation of esds in distances, angles and torsion angles; correlations between esds in cell parameters are only used when they are defined by crystal symmetry. An approximate (isotropic) treatment of cell esds is used for estimating esds involving l.s. planes.
Refinement. Refinement of F^2^ against ALL reflections. The weighted R-factor wR and goodness of fit S are based on F^2^, conventional R-factors R are based on F, with F set to zero for negative F^2^. The threshold expression of F^2^ > 2sigma(F^2^) is used only for calculating R-factors(gt) etc. and is not relevant to the choice of reflections for refinement. R-factors based on F^2^ are statistically about twice as large as those based on F, and R- factors based on ALL data will be even larger.

### Fractional atomic coordinates and isotropic or equivalent isotropic displacement parameters (Å^2^)

**Table:**

	*x*	*y*	*z*	*U*_iso_*/*U*_eq_	
F1	−0.2892 (3)	0.4118 (2)	1.37589 (19)	0.1691 (12)	
F2	−0.2201 (3)	0.26170 (19)	1.45438 (13)	0.1348 (9)	
F3	0.1983 (3)	−0.00133 (18)	1.41169 (13)	0.1326 (9)	
F4	0.1523 (2)	−0.09493 (19)	1.32178 (16)	0.1239 (7)	
O1	0.7795 (2)	0.64315 (15)	0.59135 (11)	0.0854 (6)	
O2	0.9274 (2)	0.45986 (15)	0.59554 (11)	0.0740 (5)	
O3	0.48397 (19)	0.29964 (14)	0.91556 (11)	0.0664 (5)	
O4	−0.1729 (2)	0.28577 (17)	1.32169 (14)	0.0956 (7)	
O5	1.4711 (2)	0.25405 (15)	0.60847 (12)	0.0726 (5)	
O6	1.3643 (2)	0.06175 (15)	0.61500 (12)	0.0774 (5)	
O7	0.98744 (19)	0.24742 (14)	0.91141 (12)	0.0718 (5)	
O8	0.2832 (2)	0.05283 (16)	1.28611 (12)	0.0853 (6)	
N1	0.5169 (2)	0.48188 (16)	0.84610 (12)	0.0561 (5)	
H1A	0.4881	0.5502	0.8459	0.067*	
N2	0.3146 (2)	0.55824 (16)	0.95026 (12)	0.0539 (5)	
N3	0.2702 (2)	0.37827 (15)	1.01521 (12)	0.0528 (5)	
N4	0.18439 (19)	0.45224 (15)	1.04774 (11)	0.0494 (5)	
N5	1.0411 (2)	0.10370 (16)	0.84462 (12)	0.0575 (5)	
H5A	1.0182	0.0360	0.8443	0.069*	
N6	0.7726 (2)	0.11814 (15)	1.00419 (12)	0.0549 (5)	
N7	0.8388 (2)	−0.02784 (16)	0.94376 (12)	0.0544 (5)	
N8	0.6917 (2)	0.02766 (15)	1.03503 (12)	0.0501 (5)	
C1	0.6230 (2)	0.46897 (19)	0.78493 (14)	0.0504 (5)	
C2	0.6469 (3)	0.56221 (19)	0.71933 (15)	0.0570 (6)	
H2A	0.5932	0.6289	0.7175	0.068*	
C3	0.7485 (3)	0.5570 (2)	0.65761 (15)	0.0578 (6)	
C4	0.8284 (3)	0.4564 (2)	0.65991 (15)	0.0559 (6)	
C5	0.8042 (3)	0.3651 (2)	0.72421 (15)	0.0575 (6)	
H5B	0.8575	0.2982	0.7258	0.069*	
C6	0.7021 (3)	0.3699 (2)	0.78718 (15)	0.0554 (6)	
H6A	0.6872	0.3070	0.8304	0.067*	
C7	0.7015 (5)	0.7467 (2)	0.5852 (2)	0.1142 (14)	
H7A	0.7275	0.7977	0.5335	0.171*	
H7B	0.7226	0.7791	0.6320	0.171*	
H7C	0.6015	0.7337	0.5859	0.171*	
C8	1.0015 (3)	0.3576 (2)	0.58939 (17)	0.0751 (8)	
H8A	1.0621	0.3704	0.5398	0.113*	
H8B	0.9343	0.3017	0.5859	0.113*	
H8C	1.0583	0.3315	0.6383	0.113*	
C9	0.4546 (2)	0.4004 (2)	0.90504 (14)	0.0508 (6)	
C10	0.3447 (2)	0.44616 (18)	0.95750 (14)	0.0478 (5)	
C11	0.2133 (3)	0.55853 (19)	1.00804 (15)	0.0534 (6)	
H11A	0.1679	0.6232	1.0198	0.064*	
C12	0.0877 (2)	0.41099 (19)	1.11610 (14)	0.0513 (6)	
C13	0.0736 (3)	0.2964 (2)	1.1441 (2)	0.0861 (10)	
H13A	0.1224	0.2458	1.1167	0.103*	
C14	−0.0134 (4)	0.2578 (2)	1.2127 (2)	0.0979 (11)	
H14A	−0.0225	0.1805	1.2325	0.117*	
C15	−0.0870 (3)	0.3319 (2)	1.25244 (17)	0.0681 (7)	
C16	−0.0769 (3)	0.4452 (2)	1.22283 (16)	0.0641 (7)	
H16A	−0.1295	0.4957	1.2486	0.077*	
C17	0.0116 (3)	0.4850 (2)	1.15448 (15)	0.0574 (6)	
H17A	0.0194	0.5624	1.1344	0.069*	
C18	−0.1793 (4)	0.3348 (3)	1.3868 (2)	0.0925 (10)	
H18A	−0.0892	0.3683	1.3946	0.111*	
C19	1.1504 (2)	0.14830 (19)	0.78631 (14)	0.0526 (6)	
C20	1.2112 (3)	0.2503 (2)	0.78470 (16)	0.0573 (6)	
H20A	1.1817	0.2933	0.8234	0.069*	
C21	1.3170 (3)	0.2879 (2)	0.72463 (16)	0.0575 (6)	
H21A	1.3563	0.3573	0.7228	0.069*	
C22	1.3648 (3)	0.2252 (2)	0.66795 (15)	0.0557 (6)	
C23	1.3058 (3)	0.1200 (2)	0.67165 (15)	0.0571 (6)	
C24	1.1994 (3)	0.0835 (2)	0.72955 (15)	0.0583 (6)	
H24A	1.1593	0.0144	0.7310	0.070*	
C25	1.4982 (3)	0.3698 (2)	0.58133 (19)	0.0810 (9)	
H25A	1.5685	0.3797	0.5364	0.122*	
H25B	1.5327	0.3976	0.6275	0.122*	
H25C	1.4118	0.4108	0.5616	0.122*	
C26	1.3106 (4)	−0.0464 (3)	0.6163 (2)	0.0979 (11)	
H26A	1.3612	−0.0794	0.5749	0.147*	
H26B	1.2111	−0.0384	0.6039	0.147*	
H26C	1.3232	−0.0944	0.6711	0.147*	
C27	0.9680 (3)	0.1532 (2)	0.90072 (15)	0.0545 (6)	
C28	0.8581 (2)	0.08040 (19)	0.95009 (15)	0.0512 (6)	
C29	0.7337 (2)	−0.05840 (19)	0.99824 (14)	0.0523 (6)	
H29A	0.6940	−0.1291	1.0098	0.063*	
C30	0.5838 (2)	0.03304 (18)	1.09822 (14)	0.0491 (5)	
C31	0.5146 (2)	−0.06285 (19)	1.13650 (15)	0.0517 (6)	
H31A	0.5362	−0.1305	1.1200	0.062*	
C32	0.4136 (3)	−0.05910 (19)	1.19900 (15)	0.0546 (6)	
H32A	0.3664	−0.1239	1.2248	0.066*	
C33	0.3829 (3)	0.0410 (2)	1.22304 (16)	0.0614 (6)	
C34	0.4491 (3)	0.1375 (2)	1.18296 (18)	0.0736 (8)	
H34A	0.4254	0.2054	1.1985	0.088*	
C35	0.5496 (3)	0.1346 (2)	1.12029 (17)	0.0657 (7)	
H35A	0.5940	0.2001	1.0931	0.079*	
C36	0.2545 (4)	−0.0386 (3)	1.34734 (19)	0.0779 (8)	
H36A	0.3400	−0.0868	1.3631	0.093*	

### Atomic displacement parameters (Å^2^)

**Table:**

	*U*^11^	*U*^22^	*U*^33^	*U*^12^	*U*^13^	*U*^23^
F1	0.177 (2)	0.127 (2)	0.190 (3)	0.0216 (18)	0.092 (2)	−0.0273 (18)
F2	0.189 (2)	0.1205 (17)	0.0903 (14)	−0.0618 (16)	0.0480 (14)	−0.0101 (12)
F3	0.206 (2)	0.1030 (15)	0.0909 (14)	−0.0314 (15)	0.0642 (15)	−0.0315 (12)
F4	0.1062 (15)	0.1181 (17)	0.158 (2)	−0.0444 (13)	0.0259 (14)	−0.0495 (15)
O1	0.1324 (18)	0.0492 (11)	0.0658 (12)	−0.0047 (11)	0.0329 (12)	0.0012 (9)
O2	0.0851 (13)	0.0655 (12)	0.0656 (11)	−0.0040 (10)	0.0265 (10)	−0.0061 (9)
O3	0.0839 (12)	0.0448 (10)	0.0719 (11)	−0.0095 (8)	0.0226 (9)	−0.0179 (8)
O4	0.1259 (18)	0.0696 (13)	0.0957 (15)	−0.0441 (12)	0.0581 (14)	−0.0289 (12)
O5	0.0794 (12)	0.0547 (11)	0.0801 (12)	−0.0121 (9)	0.0209 (10)	−0.0082 (9)
O6	0.1040 (15)	0.0593 (11)	0.0720 (12)	−0.0199 (10)	0.0162 (11)	−0.0205 (9)
O7	0.0788 (12)	0.0438 (10)	0.0874 (13)	−0.0029 (9)	0.0128 (10)	−0.0045 (9)
O8	0.1141 (16)	0.0582 (12)	0.0792 (13)	0.0001 (11)	0.0334 (12)	−0.0117 (10)
N1	0.0673 (13)	0.0437 (11)	0.0565 (12)	−0.0032 (9)	0.0119 (10)	−0.0109 (9)
N2	0.0607 (12)	0.0472 (11)	0.0537 (11)	−0.0052 (9)	0.0059 (10)	−0.0114 (9)
N3	0.0577 (12)	0.0455 (11)	0.0571 (11)	−0.0056 (9)	0.0071 (9)	−0.0158 (9)
N4	0.0527 (11)	0.0432 (10)	0.0530 (11)	−0.0059 (8)	0.0056 (9)	−0.0120 (9)
N5	0.0611 (12)	0.0479 (11)	0.0615 (12)	−0.0102 (9)	−0.0024 (10)	−0.0054 (10)
N6	0.0558 (12)	0.0426 (11)	0.0619 (12)	−0.0032 (9)	−0.0024 (10)	−0.0017 (9)
N7	0.0546 (12)	0.0499 (12)	0.0565 (12)	0.0000 (9)	−0.0048 (10)	−0.0063 (9)
N8	0.0528 (11)	0.0419 (10)	0.0529 (11)	−0.0017 (8)	−0.0059 (9)	−0.0036 (9)
C1	0.0587 (14)	0.0465 (13)	0.0473 (13)	−0.0077 (11)	0.0043 (11)	−0.0127 (10)
C2	0.0749 (16)	0.0417 (13)	0.0545 (14)	−0.0034 (11)	0.0065 (12)	−0.0120 (11)
C3	0.0777 (17)	0.0454 (13)	0.0486 (13)	−0.0115 (12)	0.0062 (12)	−0.0062 (11)
C4	0.0612 (15)	0.0565 (15)	0.0495 (13)	−0.0098 (12)	0.0090 (11)	−0.0108 (11)
C5	0.0587 (15)	0.0531 (14)	0.0573 (14)	0.0019 (11)	0.0058 (12)	−0.0062 (11)
C6	0.0598 (14)	0.0501 (14)	0.0527 (14)	−0.0061 (11)	0.0064 (12)	−0.0035 (11)
C7	0.202 (4)	0.0477 (18)	0.082 (2)	0.010 (2)	0.033 (2)	0.0023 (15)
C8	0.0755 (18)	0.082 (2)	0.0618 (16)	0.0098 (15)	0.0139 (14)	−0.0073 (14)
C9	0.0580 (14)	0.0476 (14)	0.0489 (13)	−0.0098 (11)	0.0023 (11)	−0.0144 (11)
C10	0.0529 (13)	0.0451 (13)	0.0475 (12)	−0.0090 (10)	0.0012 (11)	−0.0132 (10)
C11	0.0581 (14)	0.0435 (13)	0.0580 (14)	−0.0027 (11)	0.0024 (12)	−0.0106 (11)
C12	0.0519 (13)	0.0463 (13)	0.0562 (14)	−0.0097 (10)	0.0058 (11)	−0.0121 (11)
C13	0.107 (2)	0.0462 (15)	0.103 (2)	−0.0101 (15)	0.0483 (19)	−0.0191 (15)
C14	0.129 (3)	0.0442 (15)	0.117 (3)	−0.0235 (16)	0.063 (2)	−0.0168 (16)
C15	0.0795 (18)	0.0539 (15)	0.0723 (17)	−0.0220 (13)	0.0276 (14)	−0.0172 (13)
C16	0.0734 (17)	0.0507 (14)	0.0705 (17)	−0.0087 (12)	0.0176 (14)	−0.0201 (12)
C17	0.0700 (16)	0.0412 (13)	0.0620 (15)	−0.0099 (11)	0.0109 (13)	−0.0138 (11)
C18	0.109 (3)	0.081 (2)	0.086 (2)	−0.033 (2)	0.036 (2)	−0.0145 (19)
C19	0.0524 (13)	0.0496 (13)	0.0518 (13)	−0.0051 (11)	−0.0062 (11)	−0.0009 (11)
C20	0.0594 (15)	0.0479 (14)	0.0638 (15)	−0.0041 (11)	−0.0013 (12)	−0.0100 (11)
C21	0.0600 (15)	0.0428 (13)	0.0689 (16)	−0.0071 (11)	−0.0024 (13)	−0.0095 (12)
C22	0.0545 (14)	0.0479 (14)	0.0593 (15)	−0.0053 (11)	−0.0011 (12)	0.0003 (11)
C23	0.0689 (16)	0.0504 (14)	0.0516 (14)	−0.0052 (12)	−0.0023 (12)	−0.0096 (11)
C24	0.0660 (16)	0.0491 (14)	0.0590 (15)	−0.0141 (12)	−0.0054 (12)	−0.0066 (11)
C25	0.093 (2)	0.0598 (17)	0.087 (2)	−0.0234 (15)	0.0228 (17)	−0.0089 (15)
C26	0.139 (3)	0.070 (2)	0.095 (2)	−0.029 (2)	0.014 (2)	−0.0370 (18)
C27	0.0541 (14)	0.0440 (14)	0.0593 (15)	0.0037 (11)	−0.0050 (12)	0.0016 (11)
C28	0.0496 (13)	0.0458 (13)	0.0540 (14)	0.0027 (10)	−0.0094 (11)	−0.0017 (11)
C29	0.0576 (14)	0.0437 (13)	0.0548 (14)	−0.0019 (11)	−0.0073 (12)	−0.0081 (11)
C30	0.0547 (14)	0.0419 (12)	0.0487 (13)	0.0002 (10)	−0.0067 (11)	−0.0054 (10)
C31	0.0554 (14)	0.0397 (12)	0.0589 (14)	0.0011 (10)	−0.0069 (12)	−0.0083 (10)
C32	0.0607 (15)	0.0428 (13)	0.0574 (14)	−0.0047 (11)	−0.0011 (12)	−0.0041 (11)
C33	0.0734 (17)	0.0524 (15)	0.0572 (15)	−0.0014 (12)	0.0069 (13)	−0.0110 (12)
C34	0.102 (2)	0.0450 (14)	0.0749 (18)	−0.0055 (14)	0.0198 (16)	−0.0183 (13)
C35	0.0866 (19)	0.0410 (13)	0.0681 (16)	−0.0094 (12)	0.0084 (15)	−0.0091 (12)
C36	0.095 (2)	0.0670 (18)	0.0727 (19)	−0.0179 (16)	0.0196 (17)	−0.0168 (15)

### Geometric parameters (Å, °)

**Table:**

F1—C18	1.351 (4)	C7—H7B	0.9600
F2—C18	1.311 (3)	C7—H7C	0.9600
F3—C36	1.314 (3)	C8—H8A	0.9600
F4—C36	1.332 (3)	C8—H8B	0.9600
O1—C3	1.361 (3)	C8—H8C	0.9600
O1—C7	1.411 (4)	C9—C10	1.483 (3)
O2—C4	1.372 (3)	C11—H11A	0.9300
O2—C8	1.416 (3)	C12—C17	1.366 (3)
O3—C9	1.218 (3)	C12—C13	1.377 (3)
O4—C18	1.322 (4)	C13—C14	1.371 (4)
O4—C15	1.392 (3)	C13—H13A	0.9300
O5—C22	1.369 (3)	C14—C15	1.368 (4)
O5—C25	1.409 (3)	C14—H14A	0.9300
O6—C23	1.366 (3)	C15—C16	1.361 (3)
O6—C26	1.424 (3)	C16—C17	1.378 (3)
O7—C27	1.216 (3)	C16—H16A	0.9300
O8—C36	1.350 (3)	C17—H17A	0.9300
O8—C33	1.393 (3)	C18—H18A	0.9800
N1—C9	1.356 (3)	C19—C20	1.382 (3)
N1—C1	1.413 (3)	C19—C24	1.393 (3)
N1—H1A	0.8600	C20—C21	1.390 (3)
N2—C11	1.316 (3)	C20—H20A	0.9300
N2—C10	1.357 (3)	C21—C22	1.371 (3)
N3—C10	1.311 (3)	C21—H21A	0.9300
N3—N4	1.360 (2)	C22—C23	1.403 (3)
N4—C11	1.346 (3)	C23—C24	1.370 (3)
N4—C12	1.429 (3)	C24—H24A	0.9300
N5—C27	1.351 (3)	C25—H25A	0.9600
N5—C19	1.417 (3)	C25—H25B	0.9600
N5—H5A	0.8600	C25—H25C	0.9600
N6—C28	1.314 (3)	C26—H26A	0.9600
N6—N8	1.358 (2)	C26—H26B	0.9600
N7—C29	1.315 (3)	C26—H26C	0.9600
N7—C28	1.361 (3)	C27—C28	1.486 (3)
N8—C29	1.351 (3)	C29—H29A	0.9300
N8—C30	1.430 (3)	C30—C31	1.375 (3)
C1—C6	1.379 (3)	C30—C35	1.379 (3)
C1—C2	1.396 (3)	C31—C32	1.374 (3)
C2—C3	1.371 (3)	C31—H31A	0.9300
C2—H2A	0.9300	C32—C33	1.371 (3)
C3—C4	1.399 (3)	C32—H32A	0.9300
C4—C5	1.368 (3)	C33—C34	1.374 (4)
C5—C6	1.389 (3)	C34—C35	1.372 (4)
C5—H5B	0.9300	C34—H34A	0.9300
C6—H6A	0.9300	C35—H35A	0.9300
C7—H7A	0.9600	C36—H36A	0.9800
			
C3—O1—C7	117.8 (2)	C15—C16—H16A	120.2
C4—O2—C8	117.75 (19)	C17—C16—H16A	120.2
C18—O4—C15	118.3 (2)	C12—C17—C16	120.2 (2)
C22—O5—C25	117.4 (2)	C12—C17—H17A	119.9
C23—O6—C26	117.3 (2)	C16—C17—H17A	119.9
C36—O8—C33	118.7 (2)	F2—C18—O4	109.3 (3)
C9—N1—C1	128.4 (2)	F2—C18—F1	102.3 (3)
C9—N1—H1A	115.8	O4—C18—F1	108.2 (3)
C1—N1—H1A	115.8	F2—C18—H18A	112.2
C11—N2—C10	102.26 (19)	O4—C18—H18A	112.2
C10—N3—N4	102.11 (18)	F1—C18—H18A	112.2
C11—N4—N3	109.35 (18)	C20—C19—C24	119.4 (2)
C11—N4—C12	130.75 (19)	C20—C19—N5	123.3 (2)
N3—N4—C12	119.84 (18)	C24—C19—N5	117.3 (2)
C27—N5—C19	128.7 (2)	C19—C20—C21	119.3 (2)
C27—N5—H5A	115.6	C19—C20—H20A	120.3
C19—N5—H5A	115.6	C21—C20—H20A	120.3
C28—N6—N8	102.17 (18)	C22—C21—C20	121.6 (2)
C29—N7—C28	102.6 (2)	C22—C21—H21A	119.2
C29—N8—N6	109.58 (19)	C20—C21—H21A	119.2
C29—N8—C30	130.09 (19)	O5—C22—C21	125.5 (2)
N6—N8—C30	120.31 (18)	O5—C22—C23	115.7 (2)
C6—C1—C2	119.3 (2)	C21—C22—C23	118.8 (2)
C6—C1—N1	123.3 (2)	O6—C23—C24	125.6 (2)
C2—C1—N1	117.3 (2)	O6—C23—C22	114.5 (2)
C3—C2—C1	121.1 (2)	C24—C23—C22	119.9 (2)
C3—C2—H2A	119.5	C23—C24—C19	120.9 (2)
C1—C2—H2A	119.5	C23—C24—H24A	119.5
O1—C3—C2	125.3 (2)	C19—C24—H24A	119.5
O1—C3—C4	115.1 (2)	O5—C25—H25A	109.5
C2—C3—C4	119.5 (2)	O5—C25—H25B	109.5
C5—C4—O2	125.7 (2)	H25A—C25—H25B	109.5
C5—C4—C3	119.2 (2)	O5—C25—H25C	109.5
O2—C4—C3	115.1 (2)	H25A—C25—H25C	109.5
C4—C5—C6	121.6 (2)	H25B—C25—H25C	109.5
C4—C5—H5B	119.2	O6—C26—H26A	109.5
C6—C5—H5B	119.2	O6—C26—H26B	109.5
C1—C6—C5	119.3 (2)	H26A—C26—H26B	109.5
C1—C6—H6A	120.4	O6—C26—H26C	109.5
C5—C6—H6A	120.4	H26A—C26—H26C	109.5
O1—C7—H7A	109.5	H26B—C26—H26C	109.5
O1—C7—H7B	109.5	O7—C27—N5	125.0 (2)
H7A—C7—H7B	109.5	O7—C27—C28	121.9 (2)
O1—C7—H7C	109.5	N5—C27—C28	113.1 (2)
H7A—C7—H7C	109.5	N6—C28—N7	115.4 (2)
H7B—C7—H7C	109.5	N6—C28—C27	120.8 (2)
O2—C8—H8A	109.5	N7—C28—C27	123.7 (2)
O2—C8—H8B	109.5	N7—C29—N8	110.2 (2)
H8A—C8—H8B	109.5	N7—C29—H29A	124.9
O2—C8—H8C	109.5	N8—C29—H29A	124.9
H8A—C8—H8C	109.5	C31—C30—C35	120.4 (2)
H8B—C8—H8C	109.5	C31—C30—N8	119.9 (2)
O3—C9—N1	124.9 (2)	C35—C30—N8	119.7 (2)
O3—C9—C10	122.1 (2)	C32—C31—C30	120.2 (2)
N1—C9—C10	113.0 (2)	C32—C31—H31A	119.9
N3—C10—N2	115.7 (2)	C30—C31—H31A	119.9
N3—C10—C9	120.8 (2)	C33—C32—C31	119.4 (2)
N2—C10—C9	123.5 (2)	C33—C32—H32A	120.3
N2—C11—N4	110.6 (2)	C31—C32—H32A	120.3
N2—C11—H11A	124.7	C32—C33—C34	120.3 (2)
N4—C11—H11A	124.7	C32—C33—O8	123.5 (2)
C17—C12—C13	120.2 (2)	C34—C33—O8	116.2 (2)
C17—C12—N4	120.1 (2)	C35—C34—C33	120.7 (2)
C13—C12—N4	119.7 (2)	C35—C34—H34A	119.6
C14—C13—C12	119.1 (3)	C33—C34—H34A	119.6
C14—C13—H13A	120.5	C34—C35—C30	118.9 (2)
C12—C13—H13A	120.5	C34—C35—H35A	120.6
C15—C14—C13	120.7 (3)	C30—C35—H35A	120.6
C15—C14—H14A	119.6	F3—C36—F4	105.4 (3)
C13—C14—H14A	119.6	F3—C36—O8	107.1 (2)
C16—C15—C14	120.1 (2)	F4—C36—O8	109.0 (3)
C16—C15—O4	122.8 (2)	F3—C36—H36A	111.7
C14—C15—O4	117.0 (2)	F4—C36—H36A	111.7
C15—C16—C17	119.7 (2)	O8—C36—H36A	111.7
			
C10—N3—N4—C11	−0.3 (2)	C15—O4—C18—F2	−157.7 (3)
C10—N3—N4—C12	−177.88 (19)	C15—O4—C18—F1	91.6 (3)
C28—N6—N8—C29	0.3 (2)	C27—N5—C19—C20	7.4 (4)
C28—N6—N8—C30	179.11 (18)	C27—N5—C19—C24	−174.0 (2)
C9—N1—C1—C6	−14.1 (4)	C24—C19—C20—C21	2.1 (3)
C9—N1—C1—C2	165.7 (2)	N5—C19—C20—C21	−179.4 (2)
C6—C1—C2—C3	−0.5 (4)	C19—C20—C21—C22	−1.4 (4)
N1—C1—C2—C3	179.7 (2)	C25—O5—C22—C21	−24.7 (4)
C7—O1—C3—C2	0.1 (4)	C25—O5—C22—C23	157.9 (2)
C7—O1—C3—C4	179.5 (3)	C20—C21—C22—O5	−178.0 (2)
C1—C2—C3—O1	−180.0 (2)	C20—C21—C22—C23	−0.6 (4)
C1—C2—C3—C4	0.6 (4)	C26—O6—C23—C24	−0.2 (4)
C8—O2—C4—C5	7.0 (4)	C26—O6—C23—C22	179.1 (2)
C8—O2—C4—C3	−173.7 (2)	O5—C22—C23—O6	0.3 (3)
O1—C3—C4—C5	−179.9 (2)	C21—C22—C23—O6	−177.4 (2)
C2—C3—C4—C5	−0.4 (4)	O5—C22—C23—C24	179.6 (2)
O1—C3—C4—O2	0.7 (3)	C21—C22—C23—C24	1.9 (4)
C2—C3—C4—O2	−179.8 (2)	O6—C23—C24—C19	178.0 (2)
O2—C4—C5—C6	179.4 (2)	C22—C23—C24—C19	−1.3 (4)
C3—C4—C5—C6	0.1 (4)	C20—C19—C24—C23	−0.7 (4)
C2—C1—C6—C5	0.2 (3)	N5—C19—C24—C23	−179.4 (2)
N1—C1—C6—C5	180.0 (2)	C19—N5—C27—O7	−1.5 (4)
C4—C5—C6—C1	0.0 (4)	C19—N5—C27—C28	178.6 (2)
C1—N1—C9—O3	1.5 (4)	N8—N6—C28—N7	−0.1 (2)
C1—N1—C9—C10	−178.8 (2)	N8—N6—C28—C27	−179.70 (19)
N4—N3—C10—N2	0.3 (3)	C29—N7—C28—N6	−0.1 (3)
N4—N3—C10—C9	179.93 (19)	C29—N7—C28—C27	179.4 (2)
C11—N2—C10—N3	−0.2 (3)	O7—C27—C28—N6	3.7 (3)
C11—N2—C10—C9	−179.8 (2)	N5—C27—C28—N6	−176.4 (2)
O3—C9—C10—N3	−3.1 (3)	O7—C27—C28—N7	−175.8 (2)
N1—C9—C10—N3	177.2 (2)	N5—C27—C28—N7	4.1 (3)
O3—C9—C10—N2	176.5 (2)	C28—N7—C29—N8	0.3 (2)
N1—C9—C10—N2	−3.2 (3)	N6—N8—C29—N7	−0.4 (2)
C10—N2—C11—N4	0.0 (2)	C30—N8—C29—N7	−179.06 (19)
N3—N4—C11—N2	0.2 (3)	C29—N8—C30—C31	6.5 (3)
C12—N4—C11—N2	177.4 (2)	N6—N8—C30—C31	−171.98 (19)
C11—N4—C12—C17	−3.5 (4)	C29—N8—C30—C35	−173.4 (2)
N3—N4—C12—C17	173.5 (2)	N6—N8—C30—C35	8.1 (3)
C11—N4—C12—C13	177.7 (3)	C35—C30—C31—C32	−2.0 (3)
N3—N4—C12—C13	−5.3 (3)	N8—C30—C31—C32	178.12 (19)
C17—C12—C13—C14	−2.5 (5)	C30—C31—C32—C33	−0.3 (3)
N4—C12—C13—C14	176.3 (3)	C31—C32—C33—C34	2.2 (4)
C12—C13—C14—C15	0.9 (6)	C31—C32—C33—O8	−179.5 (2)
C13—C14—C15—C16	1.5 (5)	C36—O8—C33—C32	26.1 (4)
C13—C14—C15—O4	180.0 (3)	C36—O8—C33—C34	−155.6 (3)
C18—O4—C15—C16	−39.6 (5)	C32—C33—C34—C35	−2.0 (4)
C18—O4—C15—C14	141.9 (4)	O8—C33—C34—C35	179.6 (2)
C14—C15—C16—C17	−2.3 (5)	C33—C34—C35—C30	−0.2 (4)
O4—C15—C16—C17	179.3 (3)	C31—C30—C35—C34	2.2 (4)
C13—C12—C17—C16	1.7 (4)	N8—C30—C35—C34	−177.9 (2)
N4—C12—C17—C16	−177.1 (2)	C33—O8—C36—F3	159.3 (3)
C15—C16—C17—C12	0.7 (4)	C33—O8—C36—F4	−87.1 (3)

### Hydrogen-bond geometry (Å, °)

**Table:**

Cg2 and Cg5 are the centroids of the C1–C6 and C19–C24 rings, respectively.

**Table:**

*D*—H···*A*	*D*—H	H···*A*	*D*···*A*	*D*—H···*A*
C20—H20A···O7	0.93	2.29	2.891 (3)	122.
C6—H6A···O3	0.93	2.32	2.895 (3)	120.
N1—H1A···N2	0.86	2.33	2.767 (3)	112.
N5—H5A···N7	0.86	2.33	2.776 (3)	112.
C11—H11A···O7^i^	0.93	2.51	3.406 (3)	162.
C17—H17A···O7^i^	0.93	2.26	3.190 (3)	174.
C8—H8A···O1^ii^	0.96	2.59	3.533 (3)	167.
C31—H31A···O3^iii^	0.93	2.27	3.169 (3)	163.
C8—H8C···Cg5	0.96	2.74	3.615 (3)	152
C25—H25B···Cg2^iv^	0.96	2.68	3.578 (3)	157

Symmetry codes: (i) −*x*+1, −*y*+1, −*z*+2; (ii) −*x*+2, −*y*+1, −*z*+1; (iii) −*x*+1, −*y*, −*z*+2; (iv) *x*+1, *y*, *z*.
